# Selective Internal Radiation Therapy (SIRT) for Hepatocellular Carcinoma: Real-World Experience from a Tertiary Care Centre

**DOI:** 10.3390/jcm15041582

**Published:** 2026-02-17

**Authors:** I. Ergenc, M. Guerra Veloz, M. Seager, N. Heraghty, N. Kibriya, J. Green, A. Koundouraki, S. Selemani, K. Menon, R. Miquel, P. Ross, P. Peddu, A. Suddle

**Affiliations:** 1 Department of Hepatology, Institute of Liver Studies, King’s College Hospital, London SE5 9RS, UK; ilkay.ergenc@nhs.net (I.E.);; 2 Department of Radiology, King’s College Hospital, London SE5 9RS, UK; 3 Department of Nuclear Medicine, King’s College Hospital, London SE5 9RS, UK; 4 Department of Liver Transplant Surgery, Institute of Liver Studies, King’s College Hospital, London SE5 9RS, UK; 5 Department of Histology, Institute of Liver Studies, King’s College Hospital, London SE5 9RS, UK; 6 Department of Medical Oncology, King’s College Hospital, London SE5 9RS, UK

**Keywords:** hepatocellular carcinoma, prognostic factors, selective internal radiation therapy, survival, yttrium-90, radioembolization

## Abstract

**Background:** Selective internal radiation therapy (SIRT) with yttrium-90 microspheres has become an established locoregional treatment for hepatocellular carcinoma (HCC). Nevertheless, real-world data on clinical outcomes, including efficacy, safety, and prognostic determinants, remain limited. **Methods:** This study retrospectively analysed 56 patients with radiologically and/or histologically confirmed HCC who underwent SIRT at a tertiary referral centre. Baseline demographics, clinical information, tumour characteristics, procedural data, and follow-up outcomes were recorded. The primary endpoints were overall survival (OS) and progression-free survival (PFS). Secondary outcomes included radiological response (mRECIST), histological necrosis, and treatment-related toxicity. Prognostic pathways were explored using structural equation modelling (SEM). **Results:** The mean age at the beginning of SIRT was 65.0 ± 11.6 years; most patients were male (87.5%) and had preserved liver function (mean ALBI −2.9 ± 0.4). BCLC staging distribution was 50% stage A, 32.1% stage B, and 17.9% stage C. According to mRECIST criteria at 6 months, 15.2% achieved complete response (CR), 47.8% partial response (PR), 30% stable disease (SD), and 7% progressive disease (PD). Median OS was 19 months (12–32) for BCLC stage A, 28 months (3–42) for stage B, and 19 months (12–56) for stage C (log-rank *p* = 0.743). SEM identified diffuse tumour morphology as the most significant predictor of poor prognosis. Radical treatments were performed in 28% of patients, including four liver transplants and ten resections. Adverse events occurred in 11 patients, of which 7 were Clavien–Dindo grade I and 4 were grade II. **Conclusions:** In this real-world HCC group, SIRT provided durable tumour control and survival with excellent tolerability.

## 1. Introduction

Hepatocellular carcinoma (HCC) is the most common primary malignancy of the liver and the third leading cause of cancer-related death worldwide [1,2]. Global rates continue to rise, mainly driven by the evolving epidemiology of metabolic dysfunction-associated steatotic liver disease (MASLD) and alcohol-related liver disease as well as ongoing chronic viral hepatitis burden [3]. Despite improved screening and awareness, many patients present with intermediate or advanced disease stages, for which locoregional and systemic therapies are required [4].

Selective internal radiation therapy (SIRT), also termed trans-arterial radioembolization (TARE), has emerged over the past two decades as an important treatment modality for HCC. The technique delivers targeted radiotherapy by selectively delivering high doses of intra-arterial yttrium-90 (Y-90) microspheres to tumour tissue [5]. SIRT is technically versatile and applicable across the whole spectrum of the disease, from early to advanced disease, including patients with portal vein thrombosis (PVT), where other locoregional modalities such as trans-arterial chemoembolization (TACE) may be contraindicated [6,7].

Clinical trials have demonstrated high tumour response rates and durable local disease control, particularly in early and intermediate stages of HCC [5,8,9,10,11,12,13]. Consequently, SIRT has been approved and incorporated into international guidelines, confirming its role as a key standard locoregional treatment alongside ablation, resection, and TACE [14,15].

Furthermore, SIRT can be used not only in unresectable or inoperable cases but also as a downstaging or bridging strategy to curative therapies such as resection and transplantation [6,16,17]. More recently, attention has shifted towards integrating SIRT into multimodal treatment paradigms, advancing personalised HCC management, optimising dosimetry, and improving long-term outcomes [6,11,16,18,19,20,21]. The greatest survival benefits are achieved when SIRT is delivered through multidisciplinary teamwork at specialised centres [22]. Nevertheless, the subgroup of patients most likely to benefit from SIRT, as well as the optimal dosimetry, has yet to be clearly defined. In the present study, we aimed to analyse the clinical, radiological, and histological efficacy and safety of SIRT in a real-world cohort of patients with HCC treated through multidisciplinary teamwork at a tertiary referral centre.

## 2. Methods

### 2.1. Study Design and Patient Population

**Figure 1:**
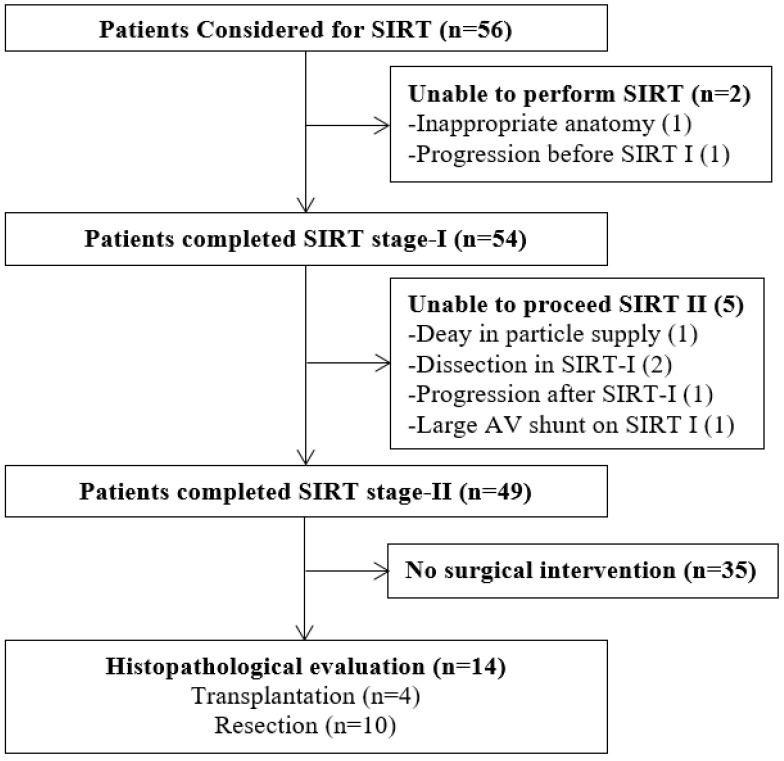
Flowchart of enrolled patients. Stage I (work-up) involves angiographic mapping, embolization of extrahepatic vessels, and shunt assessment, followed by Stage II with intra-arterial delivery of radioactive microspheres for targeted tumour irradiation.

This retrospective cohort study analysed 56 patients with HCC who were treated with SIRT between February 2019 and September 2023 at King’s College Hospital. Inclusion criteria were age ≥ 18 years, HCC confirmed according to Liver Imaging Reporting and Data System (LI-RADS) criteria [23], and liver function suitable for SIRT [21]. For non-cirrhotic patients, lesional biopsy was performed, when necessary, based on standard diagnostic recommendations for HCC [15,24]. Patients with a history of liver transplantation, concurrent non-HCC malignancies, or incomplete data were excluded. Figure 1 presents a flow diagram of patient enrolment, exclusions, and the final study cohort. This study was conducted in accordance with the Declaration of Helsinki. Research Ethics Committee approval and individual informed consent were waived in accordance with UK Health Research Authority guidance governing retrospective regional service evaluations using anonymised patient data.

### 2.2. Baseline Assessments

Demographic, clinical, and laboratory data were obtained from institutional electronic health records. Baseline prognostic liver functions were assessed by albumin–bilirubin (ALBI) score [25], the Model for End-Stage Liver Disease (MELD) [26], and the United Kingdom Model for End-Stage Liver Disease (UKELD) [27]. Cirrhosis stage was evaluated using the Child–Turcotte–Pugh (CTP) classification [28] and functional status was assessed with the Eastern Cooperative Oncology Group (ECOG) performance score. Two experienced hepatobiliary interventional radiologists staged tumours using cross-sectional imaging, the Barcelona Clinic Liver Cancer (BCLC) system [4] and the American Joint Committee on Cancer (AJCC) tumour–node–metastasis (TNM) classification [29]. Recorded tumour characteristics included size, number, presence of a diffuse pattern, vascular invasion, satellite lesions, and LI-RADS category. The extent of portal vein invasion was classified according to the Japanese Liver Cancer Study Group [30] vascular portal (VP) system, where VP1 indicates segmental or more peripheral branch invasion, VP2 involvement of second-order branches, VP3 invasion of the right or left portal vein, and VP4 involvement of the thrombus extending to the main trunk or contralateral branch.

### 2.3. Selective Internal Radiation Therapy (SIRT)

All patients underwent pre-procedural angiographic evaluation to assess vascular anatomy and identify any extrahepatic shunting. The liver shunt fraction was calculated using Technetium-99m macroaggregated albumin single photon emission computed tomography (SPECT, Siemens, Erlangen, Germany) or SPECT-CT scans. Following international guidelines, SIRT was delivered using Y-90 resin (SIR-Spheres, Sirtex Medical, Woburn, MA, USA) or glass microspheres (TheraSphere, Boston Scientific, Marlborough, MA, USA) [24]. Dosimetry was determined using empirical, single-compartment, or multi-compartment models, depending on tumour characteristics and institutional protocols. The primary vascular access sites were the right or left common femoral artery, with the left radial artery used less frequently. Target liver volume and intended radiation dose were calculated by the physicist prior to the procedure. Patients underwent Y-90 positron emission tomography or bremsstrahlung SPECT-CT after delivery of the microspheres. Procedural parameters, including administered activity (GBq), liver volume, delivered dose (Gy), and whether the intended dose was achieved, were recorded during treatment.

### 2.4. Treatment History and Follow-Up

Pre- and post-SIRT treatments, including ablation, TACE, systemic therapies, and curative interventions such as surgical resection or liver transplantation, were documented. Imaging follow-up was performed every 3–6 months as per standard recommendations. Radiological response was assessed in consensus by two experienced interventional hepatobiliary radiologists according to mRECIST [31]. Pathological tumour response (PTR) was assessed by an experienced liver histopathologist and expressed as the percentage of the tumour bed occupied by non-viable tissue. Histological correlation was available for a subset of patients who subsequently underwent resection or transplantation. Terminology Criteria for Adverse Events (CTCAE v5.0) [32] was used to assess toxicities.

### 2.5. Outcomes

The primary endpoints of this study were to assess the overall survival (OS) and progression-free survival (PFS), defined from the date of the first SIRT to death/progression or last follow-up. The secondary endpoints included radiological response rates and treatment-related adverse events in the whole cohort, as well as concordance between radiological response and histological necrosis in those who underwent surgical interventions, and the effects of baseline variables on outcomes were further explored through models constructed at 6 and 12 months and at final follow-up.

### 2.6. Statistical Analysis

R version 4.4.2 was used for statistical analysis, and it employs packages for data tasks. Inferential statistics were used to draw conclusions about relationships and differences between groups. Test selection was based on the normality of the numerical data (assessed with the Shapiro–Wilk test) and whether test assumptions were met. T-tests compared two independent groups of normally distributed data, and ANOVA compared more than two. For data not normally distributed, the Wilcoxon (for two groups) or the Kruskal–Wallis test (for more groups) was applied. Chi-square tests were used for categorical data when cell counts were adequate (over 5); otherwise, Fisher’s exact test was used.

Kaplan–Meier analysis was used to estimate survival outcomes, and log-rank tests were used to compare groups. Deaths occurring in the absence of documented disease progression were considered events. As the multivariate analysis was not statistically significant (Supplementary Table S1) due to the small sample size and patient heterogeneity, structural equation modelling (SEM) [33] was used to investigate the complicated links between clinical, tumour, and treatment variables. To study the effects of baseline variables on outcomes, models were built at 6 months, 12 months, and final follow-up [33]. Path coefficients, standard errors, z-values, *p*-values, and 95% confidence intervals (CIs) were reported. Goodness-of-fit indices were used to check if the model was adequate. Statistical significance was assigned when the two-sided *p*-value was <0.05.

Missing data were handled using complete case analysis, where only patients with complete records for the specific variables under investigation were included in the respective analyses. The number of patients included in each analysis is clearly indicated in the tables. While the retrospective nature of the study introduces inherent risks of information bias, we have sought to minimise selection bias by including all consecutive eligible patients identified through our institutional database.

## 3. Results

### 3.1. Baseline Characteristics

The study involved 56 patients treated with SIRT (Table 1). The screening process, exclusions, and final sample are shown in the flowchart (Figure 1). The mean age was 67.4 ± 11.4 years and 87.5% were male. The median of follow-up length was 20.5 (3–62) months. The median tumour diameter in the overall cohort was 8.2 cm (range 3.1–22.7 cm), with several patients presenting with large solitary lesions exceeding 8 cm, a subgroup that demonstrated favourable outcomes following SIRT. Of the 56 patients, 35 (62.5%) were cirrhotic. All cirrhotic patients were compensated; 29 (83%) were Child–Pugh A5 and 6 (17%) were A6. Signs of portal hypertension were observed in 33.9% of the cohort. ECOG performance status was 0 in 67.9% and 1 in 30.4% of patients. Half of the patients were BCLC stage A (14.3% with tumours < 5 cm and 35.7% with tumours ≥ 5 cm), 32.1% were stage B, and 17.9% were stage C. The most common underlying aetiology was MASLD (28.6%), followed by hepatitis C (21.4%) and hepatitis B (12.5%). The majority of patients had preserved baseline liver function, with a mean ALBI score of −2.9 ± 0.4 and a mean MELD score of 7.8 ± 1.8.

A total of 77.4% were classified as LI-RADS 5 and 20.8% as LI-RADS 4. Lesional biopsy was performed in 32 patients (57.1%); among them, 71.5% of tumours were well- or moderately differentiated. A total of 16.1% had portal vein tumour thrombus. According to the VP classification, 1 patient (11.1%) had VP1, 3 patients (33.3%) had VP2, 2 patients (22.2%) had VP3, and 3 patients (33.3%) had VP4 involvement.

**Figure 2:**
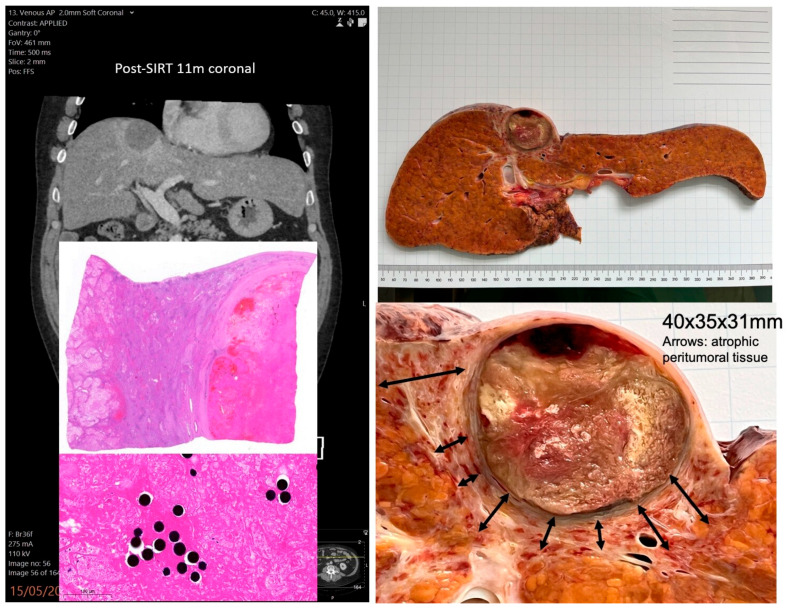
A 68-year-old man with MetALD cirrhosis and a 6 cm hepatocellular carcinoma (HCC), deemed unresectable due to cirrhosis and tumour proximity to the right portal pedicle. Treated with SIRT as a bridging therapy and underwent liver transplantation three months later.

**Figure 3:**
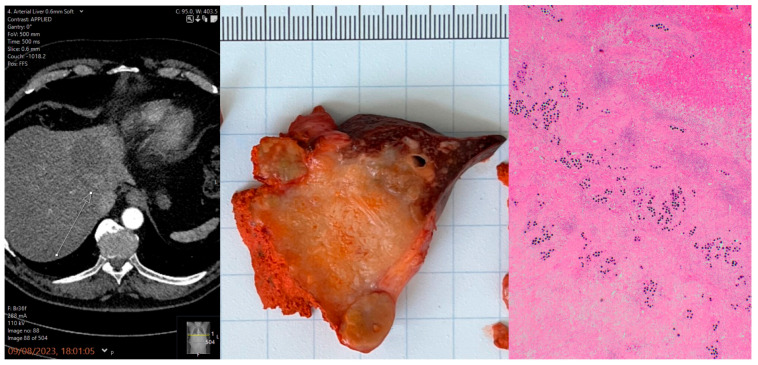
A 70-year-old man with MASLD cirrhosis and an 11 cm HCC. Treated with SIRT for tumour downstaging and underwent left hepatectomy one year later.

SIRT was the primary treatment in 67.9% of cases and was used as a first-line therapy more frequently in BCLC stage A than in stages B and C (85.7% vs. 51.7%; Table 2). Most patients received a range of treatments after SIRT, including surgery, transplantation, ablation, and systemic therapies. Example cases presented in Figure 2 and Figure 3.

### 3.2. Procedural Characteristics

A total of 27 patients received SIR-Spheres, while 22 patients were treated with TheraSpheres. Cone beam CT was used in roughly two-thirds of procedures, with similar rates between groups. Right femoral access was the most common route in both SIRT sessions across groups. Single-compartment dosimetry was predominantly employed, used in all TheraSphere patients and 70.4% of those receiving SIR-Spheres. Procedural parameters are summarised in Table 3.

### 3.3. Treatment Outcomes Stratified by BCLC Stage

**Figure 4:**
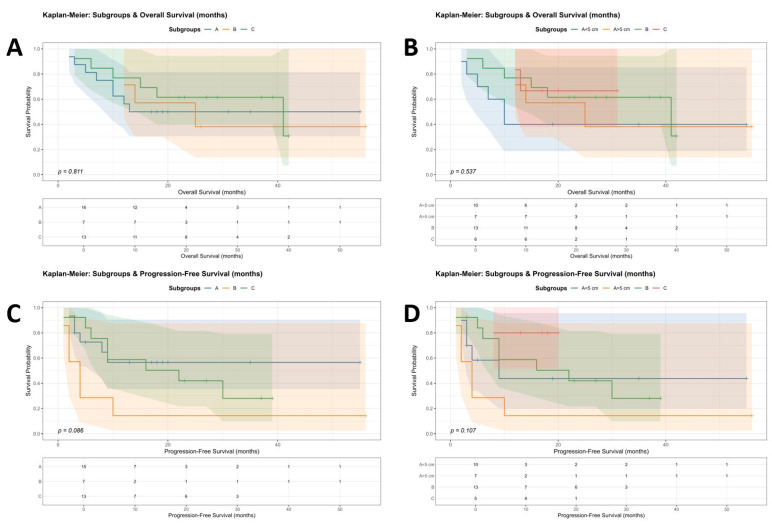
Kaplan–Meier survival curves according to BCLC stage. (**A**) Overall survival stratified by BCLC stage (IA, IB, and IC). (**B**) Overall survival stratified by BCLC stage, with subgrouping of stage A by tumour size (≤5 cm vs. >5 cm). (**C**) Progression-free survival for all patients, stratified by BCLC stage. (**D**) Progression-free survival excluding patients who received definitive treatment, stratified by BCLC stage.

Clinical characteristics, aetiology, baseline liver function, and performance status were similar across BCLC stage groups, except that cirrhosis was less common among patients with stage A tumours > 5 cm (*p* = 0.014). Overall survival did not differ significantly across BCLC stages. The median OS was 19 months (95% CI 12–32), 19 months (95% CI 2–61), 28 months (95% CI 3–42) and 19 months (95% CI 12–56) for BCLC stage A < 5 cm, BCLC stage A > 5 cm, BCLC stage B and BCLC stage C respectively (*p* = 0.743). Progression-free survival tended to be longer in BCLC A (<5 cm and >5 cm) and B groups (17.5, 16, and 19 months, respectively) than in BCLC C group (4 months), but this difference was not statistically significant (*p* = 0.192). Among patients who did not receive curative interventions, the median OS was approximately 30–32 months across all BCLC stages (*p* = 0.822) (Figure 4). Four BCLC A patients within the Milan criteria were ineligible for curative options due to malignant and cardiovascular comorbidities and died during follow-up.

### 3.4. Comparison of Radiological Response by mRECIST with Tumour Viability on Histology

Radical treatments were administered to 28% (14) of patients, including four liver transplants and ten surgical resections. The median interval between the last imaging and surgery was one month (range: 0–3 months). According to mRECIST criteria, one patient achieved complete remission (CR), two had stable disease (SD), and the remaining ten demonstrated a partial response (PR). Complete pathological tumour response (PTR; 100% necrosis) was observed only in the patient with CR. In patients with PR, PTR was heterogeneous, ranging from 19% to 98%. Among the ten patients who underwent surgical resection, six (50%) showed tumour regression of less than 50%, two had regression between 50 and 89%, and the remaining two had regression exceeding 90%. No significant correlation was observed between mRECIST-assessed radiological response and histological tumour necrosis or viable tumour (*p* = 0.473) (Table 4).

### 3.5. Radiological Response on Consecutive Follow-Up Imaging

At the first follow-up at 3 months, 47.8% (14) of patients demonstrated a partial response (PR), and 15.2% achieved a complete response (CR) radiologically by mRECIST. By the third and fourth follow-ups (12–18 months), CR rates increased to 20.0% and 33.3%, respectively, with a further reduction in median tumour diameter. Supplementary Table S2 presents radiological response over time based on serial imaging. These dynamic changes in treatment response are also illustrated in Kaplan–Meier plots as survival analysis (Figure 4).

**Figure 5:**
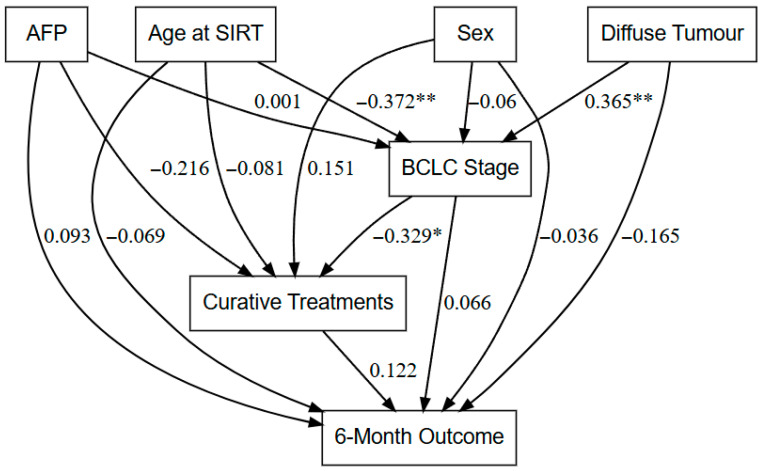
Structural equation model showing the direct and indirect effects of clinical variables on the 6-month outcome by mRECIST. Numerical values indicate standardized regression coefficients (β), representing the magnitude and direction of effects within the model, negative coefficients (−) indicate inverse associations, * *p* < 0.05; ** *p* < 0.01. AFP: Alpha-fetoprotein, SIRT: Selective internal radiation therapy, BCLC: Barcelona Clinic Liver Cancer.

Pathways influencing outcomes were explored using SEM (Figure 5; Supplementary Tables S3–S5). Across all models (6-month, 12-month, and final outcomes), the only significant path was between diffuse tumour morphology and BCLC stage (*p* = 0.006), indicating that disease stage is a key upstream determinant of prognosis. Curative treatments also demonstrated positive associations with outcomes; however, these did not reach statistical significance within the study’s follow-up period. The complexity of indirect pathways influencing long-term survival is illustrated in Supplementary Figures S1 and S2.

### 3.6. Side Effects and Complications

SIRT was generally well tolerated. Adverse events occurred in 11 (19.6%) patients, of which 7 were Clavien–Dindo grade I and 4 were grade II. Only two patients were unable to proceed to a second session due to arterial dissection during the first procedure. One patient developed a haematoma at the vascular access site, which resolved spontaneously before the second session. The most frequent adverse events were fatigue (10.7%), abdominal pain (10.7%), and nausea or vomiting (5.4%), with minimal to moderate ascites also observed in 5.4% of patients. Radiation-induced liver injury occurred in 1 patient (1.8%). A complete list of adverse events is presented in Table 5.

## 4. Discussion

This retrospective analysis of prospectively collected real-world data demonstrated that SIRT with Y-90 microspheres is an effective and safe treatment across different BCLC stages in patients with HCC. The observed median OS and PFS were similar to outcomes reported in prospective clinical trials and previous real-world studies [10,34,35]. SEM identified tumour morphology and stage as the most reliable prognostic indicators, reinforcing the growing recognition that morphological characteristics may have greater prognostic value than biomarkers in predicting survival [2,3,11,22,34]. Furthermore, this study also highlights the effectiveness of SIRT as a bridging or downstaging approach to curative treatment modalities. This finding is consistent with previous reports demonstrating up to 44% of patients being successfully downstaged, thereby enabling surgery or liver transplantation with excellent long-term survival outcomes [36]. Case series have also reported remarkable results, with SIRT converting initially inoperable tumours into respectable ones and achieving near-complete necrosis in some cases [37]. Furthermore, the use of radiation lobectomy—employing SIRT to induce contralateral hypertrophy—has facilitated resection in patients who were previously deemed inoperable [38]. Collectively, these observations highlight the evolving role of SIRT, not only as a survival-prolonging option in patients with no curative treatment options but also as a bridge to curative interventions within multidisciplinary treatment strategies.

The SARAH trial, which enrolled patients with advanced HCC randomly assigned to SIRT or sorafenib, reported a median OS of 8.0 months in the SIRT arm compared with 9.9 months in the sorafenib arm. Although SIRT did not demonstrate superiority, it was associated with better quality of life and tolerability [12]. Similarly, the SIRveNIB trial, conducted across the Asia–Pacific region, confirmed that SIRT was non-inferior to sorafenib, while showing an improved safety profile and higher radiological response rates [13]. In our cohort, 17.9% of patients were classified as BCLC stage C, and portal vein involvement was present in 16.1% of cases, including several with advanced PV3–PV4 thrombus extension. This indicates that even patients with macrovascular invasion or advanced disease were considered suitable for SIRT. Despite our cohort including a broader range of disease stages, the comparable outcomes underscore the reproducibility and external validity of these findings beyond randomised controlled settings.

In the earlier stages of disease, but with larger tumour diameters, outcomes appear particularly promising. The LEGACY trial demonstrated a remarkable 3-year OS of 86% following ablative-dose segmental SIRT in patients with solitary, unresectable tumours ≤ 8 cm [5]. Further support comes from the DOORwaY90 trial, which reported a 72% objective response rate with personalised dosimetry in patients with unresectable HCC across BCLC stages A, B, and C, including those with larger tumour burdens [34]. The favourable survival outcomes observed among patients with large solitary lesions in our study are consistent with these pivotal trials, underscoring the potential of SIRT to achieve durable disease control even in cases with higher tumour burden, with or without subsequent curative interventions.

The technical advantage of SIRT lies in its ability to deliver high doses of radiation selectively to tumour tissue while sparing the surrounding liver parenchyma, making it particularly suitable for patients with impaired hepatic function. In the TRACE trial, SIRT achieved superior tumour control and longer time to progression compared with drug-eluting bead TACE, with a comparable safety profile in patients with intermediate-stage disease [8,39]. Similarly, in the present study, favourable outcomes were observed among BCLC B patients, particularly in those who were unsuitable for or unresponsive to TACE.

In this study, histological analysis revealed a limited correlation between radiological response and pathological necrosis after SIRT. This discrepancy can be attributed to delayed necrosis, altered perfusion, and diffusion-weighted imaging artefacts commonly observed on post-SIRT CT and MRI. Although CT was used for the majority of patients in our cohort, mRECIST appears to underestimate the extent of tumour necrosis, particularly following segmental radioembolization. Similar discrepancies have been reported in large cohorts, where complete radiologic responses often did not correspond to full histologic devitalization [40,41]. Prospective trials such as DOORwaY90 confirmed this mismatch, showing extensive necrosis despite stable disease radiologically [34]. Reviews attribute this gap to delayed necrotic evolution and limitations of mRECIST criteria [6,24].

SEM analysis further enhances the understanding of treatment outcomes in the current study. The poorest prognosis was consistently associated with higher BCLC stage and diffuse tumour infiltration, which in most cases coexisted with macrovascular invasion. Notably, among the three patients with diffuse infiltration, two had concomitant portal vein tumour thrombosis (one VP3 and one VP4), suggesting that the adverse prognostic impact may be driven not only by the diffuse morphology itself but also by the presence of advanced PVT. These findings are consistent with previous research indicating that morphological characteristics, total tumour volume, and vascular invasion are more reliable predictors of survival than biochemical markers [22]. Although curative therapies such as resection or transplantation were positively associated with improved survival in our cohort, these effects did not reach statistical significance, likely reflecting the limited follow-up period after the procedures.

A subset of patients who experienced disease progression after SIRT subsequently received systemic therapies including tyrosine kinase inhibitors and immune checkpoint inhibitors and demonstrated favourable overall survival outcomes. Experimental and translational studies suggest that SIRT may modulate the tumour immune microenvironment, enhancing infiltration of cytotoxic T cells and natural killer cells, potentially increasing tumour susceptibility to immune checkpoint inhibitors [20]. Early-phase clinical trials combining SIRT with immunotherapy regimens, such as nivolumab or atezolizumab plus bevacizumab, have reported encouraging disease control rates with manageable safety profiles [6]. These findings support the consideration of SIRT within the expanding HCC treatment landscape, serving as a cytoreductive or adjunctive therapy, either before or alongside systemic treatment options.

Safety and tolerability remain key strengths of SIRT. Only two procedural complications were observed, both occurring in early cases within the cohort. Radiation-induced liver injury was reported in a single patient. Although all adverse events including mild constitutional symptoms were recorded, the overall incidence was less than one third. No patients experienced sustained hepatic decompensation, apart from three who developed mild to moderate ascites post-procedure, all of which resolved within a few weeks, and only one needed diuretic treatment. These findings are consistent with previous large studies, which reported serious adverse events in fewer than 10% of cases [41].

Advances in personalised dosimetry have markedly improved the safety and efficacy of SIRT by enabling individualised activity planning based on tumour-to-normal liver ratios. Partition- and voxel-based models allow precise dose delivery, reducing the risk of radiation-induced liver disease and enhancing tumour control [42]. Recent trials, including DOSISPHERE-01 and DOORwaY90, demonstrated that personalised dosimetry significantly increases objective response rates and overall survival compared with standard approaches [9,34]. Our findings are also consistent with recent meta-analyses and narrative reviews, which suggest that SIRT offers promising disease control and survival outcomes as a locoregional therapy for HCC [6,43].

Despite these encouraging results, several limitations in our study should be acknowledged. The retrospective design, single-centre setting, and relatively small sample size represent the primary constraints. The small sample size and heterogeneous nature of the patient cohort limited the statistical analyses, including the presence of overlapping confidence intervals in the Kaplan–Meier curves and the inability to perform Cox regression analysis to further identify risk factors. In addition, due to the study period and transitions in Nuclear Medicine departmental systems, post-SIRT dose measurements were not available for all patients, limiting the precision of dosimetry correlations. The limited number of patients and follow-up duration across different subgroups further reduce the statistical power of the analyses. Additionally, the unequal distribution of SIRT as a first-line modality across BCLC stages reflects differences in multidisciplinary team-guided treatment sequencing, based on comprehensive assessment and treatment eligibility. This introduces a possible treatment selection bias and may confound the interpretation of OS and PFS. Lastly, few of the BCLC A patients who met the Milan criteria but were unable to undergo curative treatment due to co-existing malignancies and significant cardiovascular comorbidities impacted the overall survival comparisons. Nevertheless, the consistency of our findings with larger prospective studies, the real-world nature of the data, and treatment decisions made by a multidisciplinary team strengthen their validity and underscore the clinical relevance of our observations.

## 5. Conclusions

The findings of this study reinforce the role of SIRT as a safe and effective treatment for HCC across different BCLC stages. Survival outcomes and tumour control were consistent with previous pivotal trials and real-world data, while the safety profile remained reassuring. This study highlights the efficacy of SIRT in patients with large solitary lesions and its potential role in bridging to curative treatments. Future prospective studies involving larger cohorts within specific disease subgroups, along with refined dosimetry techniques and combination strategies, will be essential to optimise and personalise the role of SIRT in HCC management.

## Figures and Tables

**Table 1:** Clinical and laboratory characteristics of study population.

	Overall, % (*n*) *n* = 56		Overall, % (*n*) *n* = 56
Age (years) *	67.4 ± 11.4	AFP (klU/L) *	14 (2–320,000)
Age at SIRT (years) *	65.0 ± 11.6	Albumin (g/L) *	42.0 ± 3.8
**Sex**		Bilirubin (mmol/L) *	10.2 ± 4.7
*Female*	12.5% (7)	CA19-9 (U/mL) *	30.5 (3–157)
*Male*	87.5% (49)	Creatinine (µmol/L) *	77.5 (46–161)
**Subgroups of BCLC staging**		INR *	1.1 ± 0.1
*A <* 5 cm	14.3% (8)	Platelet (10^9^/L) *	194,000 (45,000–573,000)
*A >* 5 cm	35.7% (20)	Sodium (mmol/L) *	138.8 ± 4.3
*B*	32.1 (18)	**Differentiation ^£^**	
*C*	17.9 (10)	*Poor*	28.6% (8)
**Aetiology**		*Moderate*	53.6% (15)
*ArLD*	7.1% (4)	*Well*	17.9% (5)
*Hep B*	12.5% (7)	**Diffuse Tumour**	5.4% (3)
*Hepatitis C*	21.4% (12)	Largest Tumour Diameter *	7 (3–31)
*MASLD*	28.6% (16)	**LI-RADS Dominant Lesion**	
*MetALD*	7.1% (4)	*LR-4*	20.8% (11)
ALBI Score *	−2.9 ± 0.4	*LR-5*	77.4% (41)
**Child–Turcotte–Pugh Score**		*LR-M*	1.8% (1)
5	51.8% (29)	**Steatotic Component**	3.6% (2)
6	10.7% (6)	**Portal Venous Involvement**	16.1% (9)
MELD Score *	7.8 ± 1.8	*VP1*	1.8% (1)
UKELD Score *	46.4 ± 3.2	*VP2*	5.4% (3)
**ECOG Performance Status**		*VP3*	3.6% (2)
*ECOG* 0	67.9% (38)	*VP4*	5.4% (3)
*ECOG* 1	30.4% (17)	**PHT Features**	32.1% (18)
*ECOG* 2	1.8% (1)	**Satellite Lesion**	5.4% (3)
		**Mortality**	
**TNM Staging**		*Survivor*	57.1% (32)
*T1bN0M0*	55.4% (31)	*Non-survivor*	42.9% (24)
*T2N0M0*	16.1% (9)	Overall Survival (months) *	21 (2–61)
*T2NxM0*	1.8% (1)	Progression-Free Survival (months) *	16 (1–61)
*T3N0M0*	10.7% (6)	**Presence of Progression**	51.0% (25)
*T4N0M0*	16.1% (9)	**Progression Type ^¥^**	
Tumour Number *	1 (1–5)	*Primary-targeted*	18.8% (3)
Total Tumour Diameter *	8.2 (3.1–22.7)	*Non-targeted or metachronous*	62.4% (10)
**Primary Treatment Modality**	67.9% (38)	*Non-targeted/metachronous + Extra-hepatic*	18.8% (3)
**Liver Transplantation Performed**	7.1% (4)	*Extra-hepatic*	30.4% (7)
Maximum Diameter of Largest Lesion *	8 (0–85)		

* Numeric variables were presented as median (minimum-maximum) or mean ± standard deviation. ^¥^ The calculation was based on 16 patients who had progression. ^£^ Tumour differentiation in histopathology. Abbreviations: AFP: Alpha-fetoprotein, ALBI: Albumin–bilirubin score, ALD: Alcohol-related liver disease, BCLC: Barcelona Clinic Liver Cancer, CA19-9: Carbohydrate antigen 19-9, ECOG: Eastern Cooperative Oncology Group, INR: International normalised ratio, LI-RADS: Liver Imaging Reporting and Data System, MASLD: Metabolic dysfunction-associated steatotic liver disease, MELD: Model for End-Stage Liver Disease, MetALD: Metabolic dysfunction-associated alcohol-related liver disease, PHT: Portal hypertension, SIRT: Selective internal radiation therapy, TNM: Tumour–node–metastasis staging, UKELD: United Kingdom Model for End-Stage Liver Disease.

**Table 2:** Baseline clinical, tumour, treatment, and outcome characteristics by BCLC stage.

	Stage A < 5 cm, n (%) n = 8	Stage A > 5 cm, n (%) n = 20	Stage B, n (%) n = 18	Stage C, n (%) n = 10	*p*
Age at SIRT initiation (years) *	64.5 ± 7.0	69.7 ± 9.0	65.6 ± 9.4	55.2 ± 17.2	0.127
**Presence of Cirrhosis**	7 (87.5)	7 (35.0)	14 (77.8)	7 (70.0)	0.014
ALBI Score *	−3.1 (−3.9–−2.6)	−2.9 (−3.3–−2.4)	−2.8 ± 0.5	−2.8 ± 0.4	0.384
**Child–Turcotte–Pugh Score**					0.421
5	6 (85.71)	7 (100.0)	10 (55.6)	6 (60.0)	
6	1 (14.3)	0 (0.0)	4 (22.2)	1 (10.0)	
MELD Score *	8.1 ± 2.5	7.8 ± 2.0	7.3 ± 1.3	8.2 ± 2.0	0.811
UKELD Score *	44.7 ± 1.5	47.0 ± 3.6	46.6 ± 2.3	46.4 ± 4.3	0.300
**ECOG Performance Status 0**	4 (50.0)	14 (70.0)	14 (77.8)	6 (60.0)	0.344
**Differentiation**					0.447
*Poor*	0 (0.0)	3 (23.1)	2 (22.2)	3 (60.0)	
*Moderate*	1 (100.0)	6 (46.2)	6 (66.7)	2 (40.0)	
*Well*	0 (0.0)	4 (30.8)	1 (11.1)	0 (0.0)	
**Non-survivors**	2 (25.0)	8 (40.0)	7 (38.9)	7 (70.0)	0.237
Overall Survival (months) *	19 (12–32)	19 (2–61)	28 (3–42)	19 (12–56)	0.743
Progression-Free Survival (months) *	17.5 (8–32)	16 (2–61)	19 (1–39)	4 (1–56)	0.192
**Presence of Progression**	1 (14.3)	9 (47.4)	9 (56.3)	6 (85.7)	0.059
**Progression Type**					0.896
*Primary-targeted*	0 (0.0)	1 (12.5)	1 (12.5)	1 (16.7)	
*Non-targeted or metachronous*	1 (100.0)	3 (37.5)	4 (50.0)	2 (33.3)	
*Non-targeted/metachronous + Extra-hepatic*	0 (0.0)	2 (25.0)	1 (12.5)	0 (0.0)	
*Extra-hepatic*	0 (0.0)	2 (25.0)	2 (25.0)	3 (50.0)	
**SIRT as Primary Treatment Modality**	6 (75.0)	18 (90.0)	8 (44.4)	6 (60.0)	**0.023**

* Numeric variables were presented as median (minimum-maximum) or mean ± standard deviation. Abbreviations: ALBI: Albumin–bilirubin score, BCLC: Barcelona Clinic Liver Cancer, ECOG: Eastern Cooperative Oncology Group, MASLD: Metabolic dysfunction-associated steatotic liver disease, MetALD: Steatotic liver disease with features of both MASLD and ALD, UKELD: United Kingdom Model for End-Stage Liver Disease.

**Table 3:** Procedural parameters of SIRT ^β^.

	SIR-Spheres, % (*n*) *n* = 27	TheraSpheres, % (*n*) * n * = 22	* p * Value
**Cone Beam CT**	63.0% (17)	72.7% (16)	0.549
Activity (GBq) *	1.6 (0.4–3.2)	2.5 (0.4–4.9)	** 0.048 **
**Access Site for SIRT I**			0.707
*R CFA*	70.4% (19)	81.8% (18)	
*L CFA*	18.5% (5)	9.1% (2)	
*L Radial*	11.1% (3)	9.1% (2)	
**Access Site for SIRT II**			0.155
*R CFA*	77.8% (21)	86.4% (19)	
*L CFA*	14.8% (4)	0.0% (0)	
*L Radial*	7.4% (2)	13.6% (3)	
**Perfused Volume (mL)**	800.0 (404.5–1352.5)	400.0 (238.0–631.0)	**0.015**
Perfused Dose (Gy) *	150.0 (90.0–200.0)	250.0 (200.0–385.0)	**<0.001**
**Dosimetry Style**			** 0.017 **
*Empirical*	11.1% (3)	0.0% (0)	
*Single-compartment*	70.4% (19)	100.0% (22)	
*Multi-compartment*	18.5% (5)	0.0% (0)	

* Numeric variables were presented as median (minimum-maximum) or mean ± standard deviation. ^β^ The study population consisted of 56 patients; however, dosimeter measurements were available for 49 participants. Abbreviations: CFA: Common femoral artery, GBq: Gigabecquerel, SIRT: Selective internal radiation therapy.

**Table 4:** Correlation analysis of mRECIST radiological response with histological tumour viability.

	CR n (%)	PR n (%)	SD n (%)	*p*-Value	Kappa
**Histological Necrosis Group**				0.473 ^a^	NA
>90% necrosis	0 (0.0)	1 (50.0)	2 (22.2)		
50–89% necrosis	1 (100.0)	0 (0.0)	3 (33.3)		
<50% necrosis	0 (0.0)	1 (50.0)	4 (44.4)		
**Total**	1 (7.1)	2 (14.3)	9 (64.3)		

^a^ Based on the Likelihood Ratio test (LR = 5.571; df = 6). Abbreviations: CR: Complete Response, df: Degrees of Freedom, LR: Likelihood Ratio, NA: Not Applicable, PR: Partial Response, SD: Stable Disease, χ^2^: Chi-square.

**Table 5:** Adverse events observed following SIRT procedures.

Adverse Events	Overall, % (n) n = 56
**Abdominal pain**	10.7% (6)
**Fatigue**	10.7% (6)
**Decreased appetite**	7.2% (4)
**Reduced general status**	7.1% (4)
**Nausea/vomiting**	5.4% (3)
**Ascites**	5.4% (3)
**Decompensation**	5.4% (3)
**Fever**	3.6% (2)
**Arterial dissection**	3.6% (2)
**Radiation-induced liver injury**	1.8 (1)
**Hypertensive attack**	1.8% (1)
**Atrial flutter/chest pain**	1.8% (1)
**Bleeding from access**	1.8% (1)
**Biliary stricture**	1.8% (1)
**Pain at insertion site**	1.8% (1)
**Pain during procedure**	1.8% (1)
**Pulmonary embolism**	1.8% (1)
**Portal vein thrombus**	1.8% (1)
**Haematoma at insertion**	1.8% (1)

## Data Availability

The data that support the findings of this study are available from the corresponding author upon reasonable request.

## Supplementary Materials

The following supporting information can be downloaded at: https://www.mdpi.com/article/10.3390/jcm15041582/s1, Table S1: Cox regression analysis for treatment response; Table S2: Evolution of Tumour Response and Diameter Measurements Over Multiple Follow-Ups; Table S3: SEM Analysis for 6-Month Outcome; Table S4: SEM Analysis for 12-month Outcome; Table S5: SEM Analysis for Final Outcome; Figure S1: Structural Equation Model Showing the Direct and Indirect Effects of Clinical Variables on 12-Month Outcome by mRECIST; Figure S2: Structural Equation Model Showing the Direct and Indirect Effects of Clinical Variables on Final Radiological Outcome by mRECIST.
