# Supplementary material for: Selective Internal Radiation Therapy (SIRT) for Hepatocellular Carcinoma: Real-World Experience from a Tertiary Care Centre
Source: J Clin Med. 2026 Feb 17;15(4):1582. doi: 10.3390/jcm15041582 (PMC12941426; doi:10.3390/jcm15041582)
Supplement: Supplementary file 1 [file jcm-15-01582-s001.zip › jcm-4100184-supplementary.pdf]

**Supplementary Table S1.** Cox regression analysis for treatment response.

| Variables in the Equation | B | SE | Wald | df | Sig. | Exp(B) | 95,0% CI for Exp(B) Lower | Upper |
|---|---|---|---|---|---|---|---|---|
| **BCLC Stage** | | | 1,240 | 2 | 0,538 | | | |
| **BCLC Stage A** | -0,637 | 0,591 | 1,163 | 1 | 0,281 | 0,529 | 0,166 | 1,683 |
| **BCLC Stage B** | -0,593 | 1,177 | 0,253 | 1 | 0,615 | 0,553 | 0,055 | 5,556 |
| **Curative treatments** | -2,614 | 1,108 | 5,565 | 1 | 0,018 | 0,073 | 0,008 | 0,643 |
| **Cirrhosis** | -0,603 | 0,614 | 0,965 | 1 | 0,326 | 0,547 | 0,164 | 1,823 |
| **Disease progression** | 1,433 | 0,628 | 5,212 | 1 | 0,022 | 4,191 | 1,225 | 14,344 |
| **Portal venous involvement** | -0,007 | 1,316 | 0,000 | 1 | 0,996 | 0,993 | 0,075 | 13,104 |

**Abbreviations:** B, regression coefficient; SE, standard error; Wald, Wald chi-square statistic; df, degrees of freedom; Sig., statistical significance (p value); Exp(B), exponentiated regression coefficient (hazard ratio); CI, confidence interval; BCLC, Barcelona Clinic Liver Cancer.

**Supplementary Table S2.** Evolution of Tumour Response and Diameter Measurements Over Multiple Follow-Ups

| | Overall, n (%) |
|---|---|
| **Diameter (1st FU)*** | 7.3 (1.3 - 30) |
| **Imaging Outcome (1st)** | |
| *CR* | 7 (15.2) |
| *PD* | 3 (6.5) |
| *PR* | 22 (47.8) |
| *SD* | 14 (30.4) |
| **Diameter (2nd FU)*** | 4.6 (0 - 14.7) |
| **Imaging Outcome (2nd)** | |
| *CR* | 9 (21.4) |

| | |
|---|---|
| *PD* | 5 (11.9) |
| *PR* | 21 (50.0) |
| *SD* | 7 (16.7) |
| **Diameter (3rd FU)*** | 0 (0 - 12.2) |
| **Imaging Outcome (3rd)** | |
| *CR* | 6 (20.0) |
| *PD* | 3 (10.0) |
| *PR* | 16 (53.3) |
| *SD* | 3 (10.0) |
| *Resected* | 2 (6.7) |
| **Diameter Outcome (4th)*** | 5.6 (0.8 - 11.8) |
| **Imaging Outcome (4th)** | |
| *CR* | 8 (33.3) |

| | |
|---|---|
| *PD* | 6 (25.0) |
| *PR* | 7 (29.2) |
| *SD* | 3 (12.5) |
| **Diameter Outcome (5th)*** | 5.7 (4.2 - 7.5) |
| **Imaging Outcome (5th)** | |
| *CR* | 3 (30.0) |
| *PD* | 2 (20.0) |
| *PR* | 5 (50.0) |
| **Diameter (6th FU)*** | 7.4 (5.4 - 7.8) |
| **Imaging Outcome (6th)** | |
| *CR* | 9 (75.0) |
| *PR* | 2 (16.7) |
| *SD* | 1 (8.3) |

*Numeric variables were presented as median (minimum-maximum) or mean ± standard deviation.

**Abbreviations:** CR: Complete response, FU: Follow-up, PD: Progressive disease, PR: Partial response, SD: Stable disease.

**Supplemental Table S3**. SEM Analysis for 6-Month Outcome (Model Parameter Estimates)

| Pathway | Estimate_B | Std_Estimate_beta | Std. Error | z-value | p-value | 95% CI (Lower, Upper) |
|---|---|---|---|---|---|---|
| Curative Treatments ← AFP | -0.127 | -0.216 | 0.078 | -1.635 | 0.102 | (-0.280, 0.025) |
| Curative Treatments ← BCLC Stage | -0.213 | -0.329 | 0.092 | -2.321 | 0.020 | (-0.393, -0.033) |
| Curative Treatments ← Age at SIRT | -0.004 | -0.081 | 0.006 | -0.575 | 0.566 | (-0.016, 0.009) |
| Curative Treatments ← Sex | 0.195 | 0.151 | 0.175 | 1.115 | 0.265 | (-0.148, 0.538) |
| 6-Month Outcome ← Curative Treatments | 0.248 | 0.122 | 0.327 | 0.757 | 0.449 | (-0.394, 0.889) |
| 6-Month Outcome ← BCLC Stage | 0.087 | 0.066 | 0.234 | 0.373 | 0.709 | (-0.372, 0.546) |
| 6-Month Outcome ← Diffuse Tumour | -0.628 | -0.165 | 0.633 | -0.992 | 0.321 | (-1.867, 0.612) |
| 6-Month Outcome ← AFP | 0.111 | 0.093 | 0.180 | 0.616 | 0.538 | (-0.242, 0.465) |
| 6-Month Outcome ← Age at SIRT | -0.006 | -0.069 | 0.014 | -0.440 | 0.660 | (-0.033, 0.021) |
| 6-Month Outcome ← Sex | -0.093 | -0.036 | 0.417 | -0.224 | 0.823 | (-0.911, 0.724) |
| BCLC Stage ← Diffuse Tumour | 1.060 | 0.365 | 0.383 | 2.770 | 0.006 | (0.310, 1.809) |
| BCLC Stage ← AFP | 0.001 | 0.001 | 0.114 | 0.009 | 0.993 | (-0.222, 0.224) |
| BCLC Stage ← Age at SIRT | -0.025 | -0.372 | 0.008 | -3.049 | 0.002 | (-0.042, -0.009) |
| BCLC Stage ← Sex | -0.120 | -0.060 | 0.268 | -0.449 | 0.654 | (-0.645, 0.405) |

| | | | | | | |
|---|---|---|---|---|---|---|
| **Indirect Effect (AFP)** | **0.080** | | **0.178** | **0.449** | **0.653** | **(-0.268, 0.428)** |
| **Indirect Effect (Diffuse)** | **-0.535** | | **0.587** | **-0.911** | **0.362** | **(-1.686, 0.616)** |
| **Indirect Effect (Age)** | **-0.009** | | **0.013** | **-0.713** | **0.476** | **(-0.035, 0.016)** |
| **Indirect Effect (Sex)** | **-0.055** | | **0.413** | **-0.134** | **0.893** | **(-0.865, 0.754)** |

**Abbreviations:** AFP, alpha-fetoprotein; BCLC, Barcelona Clinic Liver Cancer; SIRT, selective internal radiation therapy; Estimate_B, unstandardised regression coefficient; Std_Estimate_beta, standardised regression coefficient; Std. Error, standard error of the estimate; CI, confidence interval.

**Supplemental Table S4.** SEM Analysis for 12-month Outcome (Model Parameter Estimates)

| Pathway | Estimate_B | Std_Estimate_beta | Std. Error | z-value | p-value | 95% CI (Lower, Upper) |
|---|---|---|---|---|---|---|
| Curative Treatments ← AFP | -0.127 | -0.216 | 0.078 | -1.635 | 0.102 | (-0.280, 0.025) |
| Curative Treatments ← BCLC Stage | -0.213 | -0.329 | 0.092 | -2.321 | 0.020 | (-0.393, -0.033) |
| Curative Treatments ← Age at SIRT | -0.004 | -0.081 | 0.006 | -0.575 | 0.566 | (-0.016, 0.009) |
| Curative Treatments ← Sex | 0.195 | 0.151 | 0.175 | 1.115 | 0.265 | (-0.148, 0.538) |
| 12-Month Outcome ← Curative Treatments | 0.501 | 0.184 | 0.535 | 0.938 | 0.348 | (-0.547, 1.550) |
| 12-Month Outcome ← BCLC Stage | -0.086 | -0.049 | 0.366 | -0.236 | 0.813 | (-0.804, 0.631) |
| 12-Month Outcome ← Diffuse Tumour | -0.335 | -0.066 | 0.928 | -0.361 | 0.718 | (-2.155, 1.484) |
| 12-Month Outcome ← AFP | 0.093 | 0.058 | 0.294 | 0.315 | 0.753 | (-0.483, 0.668) |
| 12-Month Outcome ← Age at SIRT | -0.020 | -0.165 | 0.022 | -0.907 | 0.364 | (-0.062, 0.023) |
| 12-Month Outcome ← Sex | -0.652 | -0.186 | 0.683 | -0.955 | 0.340 | (-1.991, 0.686) |
| BCLC Stage ← Diffuse Tumour | 1.060 | 0.365 | 0.383 | 2.770 | 0.006 | (0.310, 1.809) |
| BCLC Stage ← AFP | 0.001 | 0.001 | 0.114 | 0.009 | 0.993 | (-0.222, 0.224) |
| BCLC Stage ← Age at SIRT | -0.025 | -0.372 | 0.008 | -3.049 | 0.002 | (-0.042, -0.009) |
| BCLC Stage ← Sex | -0.120 | -0.060 | 0.268 | -0.449 | 0.654 | (-0.645, 0.405) |
| **Indirect Effect (AFP)** | **0.029** | | **0.295** | **0.097** | **0.923** | **(-0.550, 0.607)** |

| | | | | | | |
|---|---|---|---|---|---|---|
| **Indirect Effect (Diffuse)** | **-0.427** | | **0.817** | **-0.522** | **0.601** | **(-2.028, 1.175)** |
| **Indirect Effect (Age)** | **-0.019** | | **0.021** | **-0.903** | **0.367** | **(-0.061, 0.023)** |
| **Indirect Effect (Sex)** | **-0.544** | | **0.689** | **-0.790** | **0.429** | **(-1.894, 0.806)** |

**Abbreviations:** AFP, alpha-fetoprotein; BCLC, Barcelona Clinic Liver Cancer; SIRT, selective internal radiation therapy; Estimate_B, unstandardised regression coefficient; Std_Estimate_beta, standardised regression coefficient; Std. Error, standard error of the estimate; CI, confidence interval.

**Supplemental Table S5.** SEM Analysis for Final Outcome (Model Parameter Estimates)

| Pathway | Estimate_B | Std_Estimate_beta | Std. Error | z-value | p-value | 95% CI (Lower, Upper) |
|---|---|---|---|---|---|---|
| Curative Treatments ← AFP | -0.127 | -0.216 | 0.078 | -1.635 | 0.102 | (-0.280, 0.025) |
| Curative Treatments ← BCLC Stage | -0.213 | -0.329 | 0.092 | -2.321 | 0.020 | (-0.393, -0.033) |
| Curative Treatments ← Age at SIRT | -0.004 | -0.081 | 0.006 | -0.575 | 0.566 | (-0.016, 0.009) |
| Curative Treatments ← Sex | 0.195 | 0.151 | 0.175 | 1.115 | 0.265 | (-0.148, 0.538) |
| Final Outcome ← Curative Treatments | 0.618 | 0.249 | 0.388 | 1.591 | 0.112 | (-0.143, 1.379) |
| Final Outcome ← BCLC Stage | -0.131 | -0.081 | 0.278 | -0.470 | 0.638 | (-0.675, 0.414) |
| Final Outcome ← Diffuse Tumour | -0.313 | -0.067 | 0.750 | -0.418 | 0.676 | (-1.784, 1.157) |
| Final Outcome ← AFP | 0.027 | 0.019 | 0.214 | 0.128 | 0.898 | (-0.392, 0.447) |
| Final Outcome ← Age at SIRT | -0.013 | -0.119 | 0.017 | -0.785 | 0.432 | (-0.045, 0.019) |
| Final Outcome ← Sex | -0.730 | -0.228 | 0.495 | -1.475 | 0.140 | (-1.699, 0.240) |
| BCLC Stage ← Diffuse Tumour | 1.060 | 0.365 | 0.383 | 2.770 | 0.006 | (0.310, 1.809) |
| BCLC Stage ← AFP | 0.001 | 0.001 | 0.114 | 0.009 | 0.993 | (-0.222, 0.224) |
| BCLC Stage ← Age at SIRT | -0.025 | -0.372 | 0.008 | -3.049 | 0.002 | (-0.042, -0.009) |
| BCLC Stage ← Sex | -0.120 | -0.060 | 0.268 | -0.449 | 0.654 | (-0.645, 0.405) |
| **Indirect Effect (AFP)** | **-0.051** | | **0.215** | **-0.239** | **0.811** | **(-0.473, 0.370)** |

| | | | | | | |
|---|---|---|---|---|---|---|
| **Indirect Effect (Diffuse)** | **-0.452** | | **0.697** | **-0.648** | **0.517** | **(-1.818, 0.915)** |
| **Indirect Effect (Age)** | **-0.012** | | **0.016** | **-0.756** | **0.450** | **(-0.043, 0.019)** |
| **Indirect Effect (Sex)** | **-0.594** | | **0.500** | **-1.188** | **0.235** | **(-1.573, 0.386)** |

**Abbreviations:** AFP, alpha-fetoprotein; BCLC, Barcelona Clinic Liver Cancer; SIRT, selective internal radiation therapy; Estimate_B, unstandardised regression coefficient; Std_Estimate_beta, standardised regression coefficient; Std. Error, standard error of the estimate; CI, confidence interval.

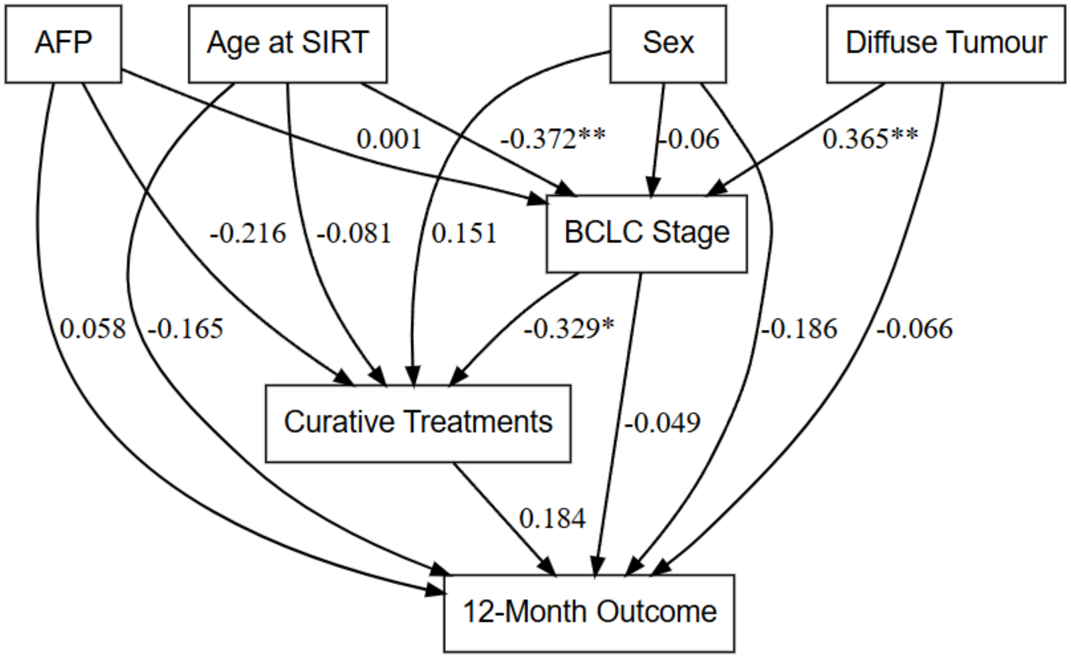


**Supplemental Figure S1.** Structural Equation Model Showing the Direct and Indirect Effects of Clinical Variables on 12-Month Outcome by mRECIST

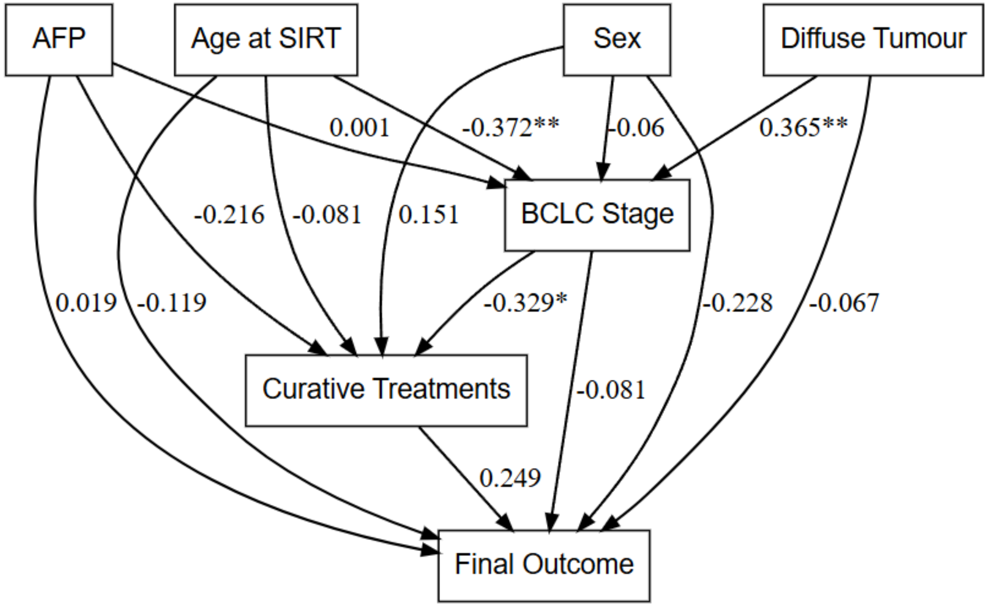


**Supplemental Figure S2.** Structural Equation Model Showing the Direct and Indirect Effects of Clinical Variables on Final Radiological Outcome by mRECIST
